# Study of Through-Hole Micro-Drilling in Sapphire by Means of Pulsed Bessel Beams

**DOI:** 10.3390/mi13040624

**Published:** 2022-04-15

**Authors:** Akhil Kuriakose, Monica Bollani, Paolo Di Trapani, Ottavia Jedrkiewicz

**Affiliations:** 1 IFN—CNR, Udr di Como, Via Valleggio 11, 22100 Como, Italy; akuriakose@studenti.uninsubria.it; 2 Dipartimento di Scienza e Alta Tecnologia, Università dell’Insubria, Via Valleggio 11, 22100 Como, Italy; paolo.ditrapani@uninsubria.it; 3 IFN—CNR, L-NESS, Via Anzani 42, 22100 Como, Italy; monica.bollani@ifn.cnr.it

**Keywords:** laser micromachining, hole drilling, Bessel beams, sapphire

## Abstract

Ultrashort Bessel beams have been used in this work to study the response of a 430-μm-thick monocrystalline sapphire sample to laser–matter interaction when injecting the beam orthogonally through the whole sample thickness. We show that with a 12° Bessel beam cone angle, we are able to internally modify the material and generate tailorable elongated microstructures while preventing the formation of surface cracks, even in the picosecond regime, contrary to what was previously reported in the literature. On the other hand, by means of Bessel beam machining combined with a trepanning technique where very high energy pulses are needed, we were able to generate 100 μm diameter through-holes, eventually with negligible cracks and very low taper angles thanks to an optimization achieved by using a 60-μm-thick layer of Kapton Polyimide removable tape.

## 1. Introduction

In this era of modern technological advancements, sapphire plays an important role in the electronic industry. Due to its physical properties such as scratch resistance, superior hardness, tensile strength, impressive thermal conductivity, chemical inertness, high melting point, thermal shock resistance, and transparency to electromagnetic waves in a wide spectral range [1], it is widely used in optoelectronic industries such as the substrate layer for light-emitting diodes [2], in integrated optical and microfluidic devices [3], and touchscreens, micromechanical devices, and optical windows [4]. Even with huge potential in consumer electronics, the power sector, and aerospace and defense applications [5], the exponential growth of sapphire usage has lagged behind expectations. One of the reasons for this is that the same advantages such as hardness that is beneficial for many applications also makes sapphire a very difficult material to fabricate very fine structures. The major problem is the crack formation during machining. In the case of laser machining, the explanation for cracks formation is based on droplet ejection during the laser irradiation [6]. Such debris continue to absorb laser energy afterward leading to higher local temperatures and high thermal stress [7]. In general, the various techniques for sapphire micromachining involve, but are not limited to, electro discharge machining (EDM), compound machining, the grinding process, and the lapping process. These processes face additional problems such as low accuracy, over time-consumption and unwanted additional damages [8].

The new center of attraction for sapphire micromachining is the use of ultrafast lasers with picosecond and femtosecond pulse durations. In [9], it was shown that sub-micrometer pit holes can be produced with single femtosecond pulses due to the self-focusing effect in air. By using a low energy fluence, microstructures without the generation of craters on the sapphire surface could be fabricated [10]. The advantage of bottom-up ablation to create less taper and crack-free holes in sapphire was also demonstrated [11]. It was also noted that there is a clear improvement in the quality of the ablated structures in multiple-shot regime as the pulse width decreases [12]. In fact, different groups have reported the same while using a standard ultrafast Gaussian beam for machining.

Recently, non-conventional beams have become widely used in ultrafast laser micromachining of transparent materials [13]. In particular, the non-diffracting nature of Bessel beams with a transverse intensity profile featured by a central narrow core and weak concentric rings [14,15] makes them a great choice for in-bulk modifications without the need for sample translation along the thickness. Indeed, thanks to their self-reconstruction property and their elongated focal zone, finite energy Bessel beams are ideal not only for internal microstructuring [16,17,18,19,20] or deep and tailorable surface modification such as microchannels for microfluidics applications [21] but also for high-impact technological applications [22] such as high-speed cutting [23,24], cleaving [25,26], welding [27] or drilling [28,29] of transparent materials.

Even though some works have demonstrated stealth dicing and cutting of sapphire using Bessel beams [25,30], the drastic usage of these for sapphire micro-structuring is limited due to crack formation and propagation, especially at the surfaces of the sample. Further, while in-bulk modifications have been reported in burst mode in the picosecond regime [31], the same using single shots, without surface cracks, has never been reported. Moreover, it has been shown in [30,32] that the crack formation depends on the pulse duration of the Bessel beam. While in the femtosecond regime, the surface cracks are less evident, their length increases for increasing pulse duration, and in the picosecond regime, three cracks in the laser written zone, especially on the top sample surface, have been observed to occur with a 120° angle between them [25,30].

In this paper, we exploit a 12° cone angle Bessel beam to induce in-bulk laser modifications in a monocrystalline c-plane sapphire sample along its whole thickness. We study the effect of the pulse duration and pulse energy and show that for a large range of these parameters, both in the femtosecond and picosecond regimes, single shot material modifications along the bulk can occur without any surface cracks in contrast to what was observed in [25,30] where the Bessel beam cone angle was greater than or equal to 20°. We also identify the picosecond regime as the best regime to obtain regular internal microstructures across the sample thickness and we study the possibility to drill holes in sapphire with picosecond Bessel pulses. This can be done by means of a trepanning-like technique [28] provided very high energy pulses are used. In this case where multiple-shot machining with hundreds of μJ pulses is adopted, the cracks appearing around the generated apertures at the surfaces can be avoided thanks to an optimization technique making use of a removable tape applied on the top surface. Its use has also been validated in a preliminary study of multiple-shot micromachining, aimed at verifying the possibility to eliminate strong radiation–matter interaction effects at the top air–sapphire interface. In this way, as shown later on, we have been able to drill 100 μm diameter through-holes, eventually with negligible cracks and a very low taper angle, which to the best of our knowledge have never been reported before for sapphire.

## 2. Materials and Methods

### 2.1. Experimental Set-Up

**Figure 1:**
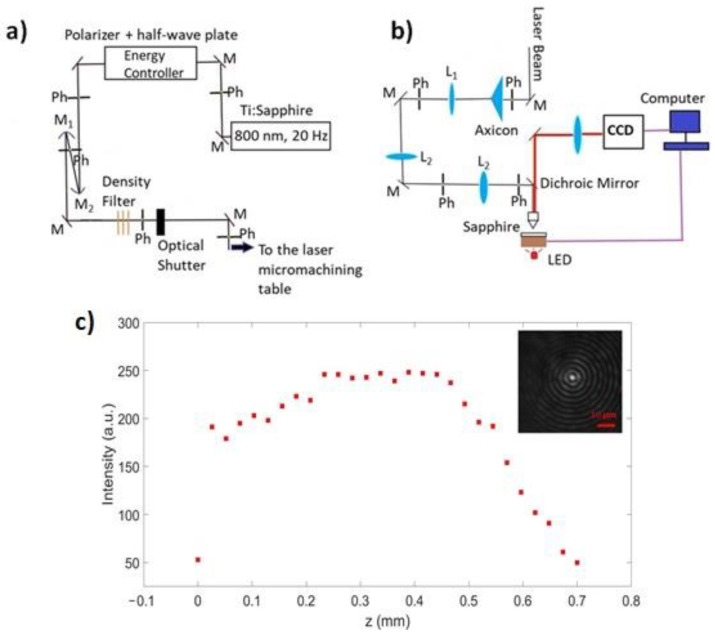
Experimental set-up for Bessel beam generation (**a**) and transparent material micromachining (**b**). In (**c**): Peak intensity evolution of the Bessel beam along the propagation direction after the microscope objective and (in the inset) transverse intensity profile recorded (over 200 ms) in the center of the Bessel zone. The intensity was evaluated by averaging the counts inside the whole central core (the vertical size of the data point reflects the statistical error).

The micromachining studies were performed by means of a 20-Hz Ti:Sapphire amplified laser system (Amplitude Technologies) delivering 40-fs transform-limited pulses at 800 nm wavelength in the mJ pulse energy range. By detuning the laser compressor, the pulse duration can be adjusted, allowing us to work in both femtosecond and picosecond regimes. The experimental set-up is shown in Figure 1. The spatially filtered Gaussian beam (5 mm beam waist after demagnification with two spherical mirrors in a telescopic configuration) is converted into a Bessel beam (BB) thanks to a fused silica axicon (178° apex angle). A telescopic system formed by a 250 mm focal length lens (L_1_) and a 0.45 N.A. 20× microscope objective (f_obj_ = 9 mm), together with a 1:1 imaging system (L_2_ lenses), allowed us to have at the sample position a final BB featured by 12° cone angle, a total central core size of about 3 μm, and a total Bessel zone of 700 μm. The transverse intensity beam profiling using a one lens imaging and a Charge-Coupled Device (CCD) camera (uEye) was carried out in air along the whole non-diffracting zone prior to micromachining (See Figure 1c). To deflect the laser beam and send it vertically towards the sample placed on a micrometer precision 3D motorized stage (driven by SCA, system control application software, Altechna Rnd, Vilnius, Lithuania), a dichroic mirror was positioned between the lens and the objective. Thanks to a backlighting Light Emitting Diode (LED) placed below the transparent sample and an imaging system using another CCD (uEye), a real-time observation of the sample surface during the micromachining process can be realized. This allows a careful adjustment of the relative positioning of the BB focal length (and thus of the core intensity profile along the propagation direction) with respect to the sample. For in-bulk machining, the Bessel zone is generally symmetrically distributed across the sample, i.e., with maximal intensity in the middle of the sample, although we try to set the beam position in such a way that a slightly lower intensity impacts the top surface with respect to the bottom. This prevents strong input interaction at the air/material interface where beam reshaping occurs because of the sudden change in the refractive index [29,33].

The pulse energy is controlled by a polarizer combined with a half waveplate and neutral density filters. Pinholes (Ph) are used to align the beam along its path and mirrors (M) are used to deflect and carry the beam towards the sample. An optical shutter, whose aperture time is controlled by software, allows us to select the number of laser pulses to send to the sample. In Figure 1b, the detailed schematic of the micromachining set-up is shown.

### 2.2. Material Sample Details

In this work, the sample used for the Bessel beam micromachining experiments is a monocrystalline c-plane sapphire of 430 μm thickness. The sample belongs to the family of hexagonal crystals. The typical crystallographic orientation of the structure is reported in [30]. There are three axes at 120 degrees to one another on the c-plane. The physical and chemical properties of sapphire are reported in Table 1 [34,35,36,37].

When working in multiple-shot mode, in order to reduce or eliminate the crack effects that can occur in this microfabrication regime, we also used a polyimide tape (Kapton Adhesive Polyimide Tape) of 60 μm thickness, whose refractive index (1.70) is comparable to that of sapphire. The Kapton layer, resistant to heat up to 350°, was applied on the top surface of the sample in order to eliminate the strong effect of radiation–matter interaction at the air–material interface where the ablation threshold is typically lower than that in the bulk, and Bessel beam reshaping and distortion can occur. This has been a key point during the study of hole drilling in sapphire. As shown later on, this allowed us to avoid crack formation around the apertures created by our Bessel beam drilling technique [28] in a multiple-shot regime featured by hundreds of μJ energy pulses.

### 2.3. Experimental Method

Our work started with a preliminary study of the single shot in-bulk modifications that can be generated along the thickness of the sapphire sample for different energies and pulse durations. The features of the internal and surface damages were observed under an optical microscope. The multiple-shot regime in view of the hole-drilling machining was also investigated, as the laser processing technique for this particular application is based on the partial superposition of neighboring Bessel beam focal lines (i.e., interaction zones in the material), with trajectories that can be concentric or spiraling. This technique is indeed based on a Bessel beam microfabrication in combination with the trepanning technique described in [28,29], without the need of sample translation along the beam propagation direction. Hole drilling in sapphire was performed with different pulse energies in the picosecond regime, and results prior to and after optimization of the aperture quality are presented. Scanning electron microscopy was used to clearly observe the resulting hole morphology.

## 3. Results and Discussion

### 3.1. Single-Shot Bessel Beam Micromachining

The length of the Bessel non-diffracting zone under our experimental conditions (700 μm in air) guaranties an interaction between the beam and our sapphire sample along its whole thickness, also in a single shot. We recall that while for a Gaussian beam the total input power is concentrated in the localized focal spot (within the Rayleigh range), for a Bessel beam it is equally distributed over the core and rings featuring the beam [38]; these act as a conical reservoir refilling the core during propagation and thus avoiding depletion effects. In turn, the beam power is distributed along the whole Bessel zone. By choosing the suitable pulse energy regime, it is thus possible to work under conditions where the central core of the pulsed BB alone will undergo nonlinear absorption along the whole focal length and, for a pulse energy above the material modification threshold, the core will be responsible for the surface damage. If the pulse energy is too high, the BB rings might contribute to the material damage as well. The preliminary microfabrication tests allowed us to establish, for our experimental conditions, the pulse energy threshold for single shot surface damage. For instance, depending on the pulse duration, this threshold value for our BB configuration was around 20 μJ for 200 fs pulses or 60 μJ for 10 ps. On the other hand, the results of the single shot micromachining investigation highlighted that only in the picosecond regime could elongated material modifications be produced within the sapphire bulk (even at pulse energies lower than the threshold values for surface damage) similar to what was previously observed in glass [20]; moreover, no top surface cracks were detected in the energy range investigated, even when using pulses of 300 μJ (the energy limit for our micromachining set-up).

**Figure 2:**
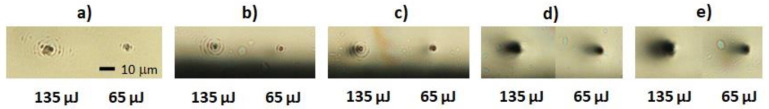
Optical microscope images of the surface traces left on the sapphire sample after single shot Bessel beam machining, for two different pulse energies and different pulse durations, namely, 200 fs (**a**), 600 fs (**b**), 1 ps (**c**), 6 ps (**d**), and 10 ps (**e**). The scale bar in (**a**) is the same for all images.

The morphology of the damage produced in a single shot at the sample surface can be observed in the images recorded under the microscope objective, reported in Figure 2 for two different pulse energies (135 μJ and 65 μJ) above the damage threshold.

The effect of a few BB rings can also be seen in the femtosecond regime in (a) and (b) and in the 1 ps regime (c). On the other hand, the beam core weakly affects the material surface, but over a larger area, when using longer pulse durations such as 6 ps or 10 ps, regimes where avalanche ionization and stronger thermal effects start to play an important role. Note that the dark halo around the damage in Figure 2d,e is due to the (out of focus) trace left on the bottom surface. No cracks departing from the central damaged zone are present, in contrast to what was observed in [30]. Nevertheless, we observed that for energies greater than 270 μJ, small cracks appeared only on the bottom surface of the sample (data not shown). We believe this may be explained by considering that the Bessel core hits the bottom surface with a higher intensity than that hitting the top, due not only to the chosen relative beam positioning with respect to the sample as previously discussed, but also due to the sudden beam reshaping at the material/air interface [29].

**Figure 3:**
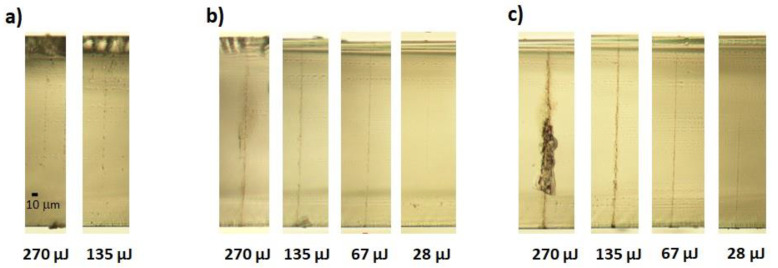
Optical microscope images. Internal modifications along the thickness of the sapphire sample reported for 1 ps (**a**), 6 ps (**b**), and 10 ps (**c**). No traces could be revealed in the fs regime or in the ps regime for lower energies. The scale bar reported in (**a**) is the same for all images.

The refractive index modifications generated inside the sapphire bulk are presented in Figure 3 for 1 ps, 6 ps, and 10 ps and for four pulse energy values. In particular, in view of hole drilling, high-energy pulses reaching 300 μJ were tested in the experiment. One can observe that the obtained microstructures were continuous only for 6 ps and 10 ps pulse durations. Nonlinear effects associated with focusing and refocusing of the BB beam due to the high intensities reached for 1 ps pulses may affect the material modifications as shown in Figure 3a. In general, in contrast to what can be observed in glass in the picosecond regime where channel-like internal modifications can be very smooth [20,28], the microstructures generated in sapphire appear to be more irregular. This could be due to the crystalline structure of sapphire and to its higher coefficient of thermal expansion (CTE) with respect to glass [29].

### 3.2. Micro-Hole Drilling in Thick Sapphire Sample

In accordance with previous drilling experiments on glass or diamond performed using Bessel beams [28,29] and with the results of our single shot tests for what concerns a most uniform and controllable energy deposition along the sapphire bulk, we mainly chose the 6 ps pulse duration regime for hole drilling in sapphire. Generally, for pulse durations between 6 and 10 ps, through-holes could be generated, but using 6 ps allowed a complete extrusion of the internal residual material, with the pulse energies available in our set-up. Femtosecond Bessel beams with a 12° cone angle did not allow us to generate holes across such thick transparent samples. With our 20 Hz Ti:Sapphire laser source and our BB geometry, the writing parameters for the realization of through-holes were featured by a fabrication speed of 0.01 mm s^−1^, corresponding to a pulse density of 2000 pulses mm^−1^, and a 0.25 μm step between the circular trajectories [28]. The number of these was set to 90 and was found to be the minimum number needed to completely extrude the material in 430-μm-thick sapphire in the available energy range. Moreover, the BB drilling process was launched three times (three-pass machining in the same position) as in thick glass or diamond [29]. Note that previous work regarding the generation of half millimeter or millimeter size holes in sapphire [7,11] was reported using standard femtosecond laser machining and vertical translation of the focal spot in a bottom-up process. In contrast, the goal of our work here was to study the possibility to generate 100-μm-size micro through-holes without any sample translation along the BB beam propagation direction and to eventually optimize the results.

#### 3.2.1. Bessel Beam Trepanning Technique Applied to Sapphire

**Figure 4:**
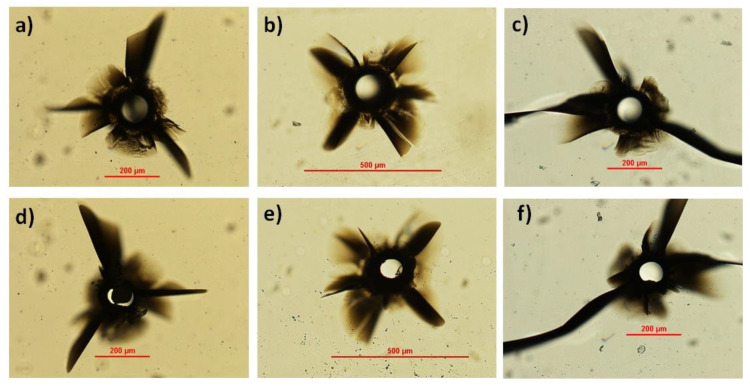
Optical microscope images of the top surface (top row) and bottom surface (bottom row) of the apertures obtained with the Bessel beam trepanning technique. Three pulse energies were considered: 230 μJ (**a**,**d**), 270 μJ (**b**,**e**), and 300 μJ (**c**,**f**). The pulse duration chosen was 6 ps.

Application of the BB trepanning technique to form through-holes in a 430-μm-thick c-plane monocrystalline sample led to the results shown in Figure 4. Evident cracks and internal lateral modifications close to the apertures at the top and bottom surfaces of the material were present, even in the case where the pulse energy was not sufficient to induce a complete extrusion of the material (see Figure 4a,d). The cracks were mostly distributed along three axes at 120 degrees with respect to the other, in accordance with the discussion on the structural characteristics of single-crystal materials [30]. These results are in contrast to those obtained in diamond, another crystalline material where the BB drilling technique was previously applied and where no cracks appeared [29]. It is worth noting that Young’s modulus of sapphire, determining its elasticity, is more than three times smaller than that of diamond, while its coefficient of thermal expansion is more than five times larger than diamond. We attribute to this the very different response of sapphire material with respect to diamond and to the stresses and plasma formation induced by the radiation–matter interaction during laser microfabrication.

**Figure 5:**
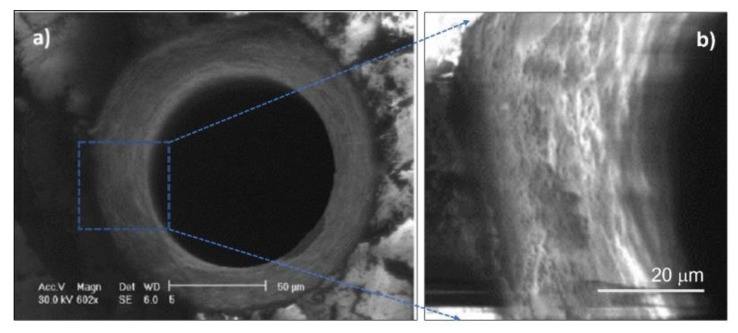
(**a**) SEM image of the aperture shown in Figure 4b obtained with the Bessel beam trepanning technique. Pulse energy 270 μJ. (**b**) Zoomed image of a portion of the aperture internal wall.

A scanning electron microscopy image of the top aperture shown in Figure 4b is presented in Figure 5a, highlighting, despite the surrounding cracks, the formation of a uniform hole. A zoomed image of the internal wall of the aperture is presented in Figure 5b.

#### 3.2.2. Removal and Reduction of Surface Cracks

**Figure 6:**
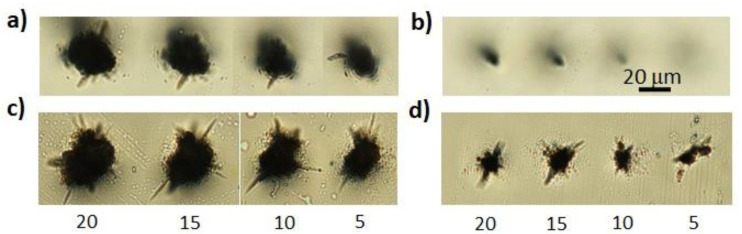
(**a**,**c**) Optical microscope images of the damage left on the top (**a**) and bottom (**c**) sapphire surfaces after multiple-shot BB machining with a pulse energy of 270 μJ and a pulse duration of 6 ps. The number of pulses used is indicated at the bottom of the figure. In (**b**,**d**) damage results of the same experiment performed by applying prior to micromachining a 60 μm layer of Kapton adhesive polyimide tape on the top of the sample. The scale bar indicated in (**b**) is the same for all images. The top surface is shown in (**b**), bottom surface in (**d**).

Given the above results, we verified the effect of multiple-shot Bessel beam machining on the sapphire sample surface, with a 6 ps pulse and pulse energy of 270 μJ. Indeed, as the BB drilling technique relies on a spiraling-like movement of the injected beam from the center (see [28] for details), there was a partial superposition of about three pulses in the same position during one circular trajectory of the laser fabrication (given the machining speed used and a 3-μm-diameter beam core). In general, considering neighboring trajectories, we should consider that in the successive circular writing trajectory, three more pulses will fall in the same area of those covered by the precedent smaller radius trajectory. Figure 6a,c show optical microscope images of the top and bottom sample surfaces hit by multiple BB shots for different number of pulses, with the laser writing parameters typically used for drilling. The typical crack formation was evident.

A complete absence of cracks on the top surface of the sapphire sample and a drastic reduction of their length on the bottom one (see Figure 6a,d) could be achieved by applying prior to micromachining a layer of Kapton adhesive polyimide tape on the sample input surface, as discussed in Section 2.2. This layer featured by the same refractive index of sapphire not only avoids the strong impact of the Bessel beam at the air–material interface where the damage threshold is typically lower than that in the bulk but also possible distortion effects of the beam when entering the material. Note that we also observed that the tape absorbs 20% of the incoming laser radiation (at 800 nm); thus, a crack length reduction in the bottom sample may be due to the reduced pulsed energy entering the sapphire bulk.

#### 3.2.3. Optimized Through-Holes in Sapphire

**Figure 7:**
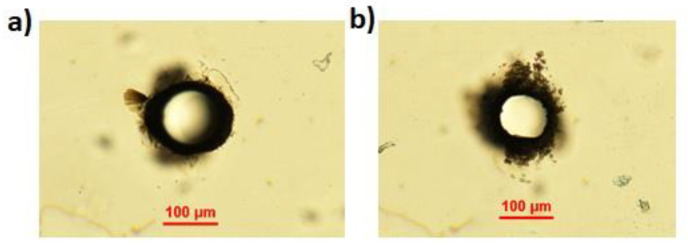
Optical microscope images of top surface (**a**) and bottom surface (**b**) of a through-hole obtained with the Bessel beam trepanning technique applied on the sapphire sample covered with a 60 μm layer of Kapton tape. The input pulse energy considered was 270 μJ with a pulse duration of 6 ps.

Following the above observations, the Bessel beam trepanning technique was again applied on the 430-μm-thick sapphire sample, this time covered with the Kapton adhesive tape. The results obtained with the same input pulse energy as before (270 μJ) and same pulse duration (6 ps) are shown in Figure 7 where we report the optical microscope images of the top and bottom sample surfaces after the micromachining. Interestingly, although the tape partially absorbed the infrared light of the BB before reaching the sapphire sample, a through-hole of about 100 μm diameter was clearly generated. In contrast to the results of Figure 4 (in particular those of Figure 4a,d considering the loss of energy due to the tape linear absorption) the aperture obtained presented no residual internal material, and only minimal stress and internal defects were observed on the top surface. A slight chipping effect, resembling that of previous observations [11], was visible on the bottom surface.

**Figure 8:**
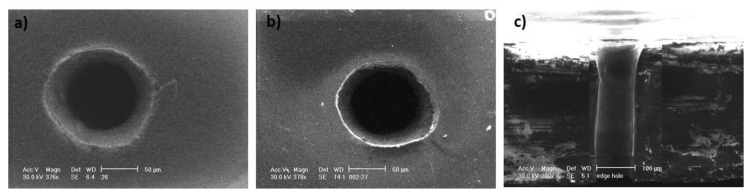
SEM image recorded after chemical etching of the top (**a**) and bottom (**b**) aperture shown in Figure 7 and obtained with the Bessel beam trepanning technique applied to the sapphire sample previously covered by a 60-μm-thick layer of Kapton tape; pulse energy 270 μJ, pulse duration 6 ps. In (**c**): SEM transverse image of a “half” through-hole drilled across the lateral edge of the sapphire sample under the same experimental conditions.

**Figure 9:**
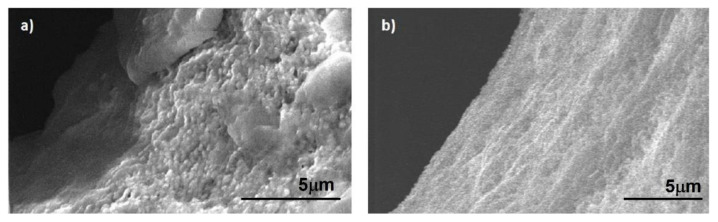
SEM images of a portion of the internal wall of the aperture shown in Figure 8a before (**a**) and after (**b**) chemical etching with hydrofluoric acid.

After chemical etching of the sapphire sample (in HF solution at 40% in volume for 24 h), we performed an SEM investigation of the top and bottom apertures generated. The recorded images are presented in Figure 8a,b, respectively, and highlight the absence of any evident stress or cracks. In Figure 8c, we present a “half” hole fabricated across the lateral edge of the material, allowing us to observe the internal morphology (in this case no chemical etching was applied) and to highlight the smoothness of the walls of the through-holes that we were able to obtain. A taper angle θ of about 5° (much lower than that of the hole of Figure 5) was evaluated from the expression

(1) $$\theta =\tan^{-1}\frac{T-B}{2h}$$

where T is the hole diameter on the top surface, B is the minimum internal hole diameter, and h is the thickness of the sample [11]. Finally, a comparison between images of the internal wall of the top aperture recorded before and after the chemical etching is presented in Figure 9.

Table 2 summarizes our results and compares the performance of our Bessel beam laser drilling technique in sapphire with that of the standard spiraling technique. The main difference is that in our work, we relied on finite energy Bessel beams to modify and ablate the internal bulk material rather than on Gaussian beams. With suitable geometry of the non-diffracting beam and the use of a Kapton tape layer on the sample top, we generated crack-less apertures on the surfaces in the picosecond regime, in contrast to previous results. More importantly, we were able to create holes much smaller than those previously presented in the literature [7,11] but with comparable taper angle. With the use of Bessel beams, the latter may be reduced when mm size holes are created. Finally note that an investigation on the use of Kapton tape together with the more standard Gaussian beam in a bottom-up machining approach may be the focus of future work.

## 4. Conclusions

The work presented here demonstrates the applicability of the pulsed Bessel beam micro-drilling technique used for sapphire for the generation of smooth holes much smaller than those previously reported in the literature and without the need of sample translation along the beam propagation direction. Single-shot micromachining first allowed us to study and analyze the internal material modification when injecting a finite energy Bessel beam orthogonally through the whole sample thickness. With a Bessel beam featured by a 12 cone angle, a 3-μm-diameter core, and a non-diffracting zone of 700 μm, we were able to generate tailorable elongated microstructures while preventing the formation of surface cracks, even in the picosecond regime. When working in multiple-shot mode, in order to reduce or eliminate the crack effects that in this case can occur in the regime needed for drilling (featured by hundreds of μJ energy pulses), we used a Kapton adhesive polyimide tape of 60 μm thickness applied on the top surface of the sample. By choosing the suitable writing conditions, we have shown the possibility to eliminate the typical stress effects of sapphire at the air–material interface where the ablation threshold is typically lower than that in the bulk and where Bessel beam reshaping and distortion can occur. This has allowed us to generate, as confirmed by SEM diagnostics, 100 μm diameter through-holes in a 430-μm-thick sapphire sample, eventually with negligible cracks, a very low taper angle, and perfectly regular internal walls.

## Figures and Tables

**Table 1:** Physical and chemical properties of sapphire.

Physical and Chemical Properties of Sapphire	
Refractive index	1.77
Hardness (on Mohs scale)	9
Density (kg/m^3^)	3.98 × 10^3^
CTE (10^−6^ K^−1^)	5.5
Young modulus Y (GPa)	345
Melting temperature (°C)	2040
Thermal Conductivity (W/m·K)	23–25
Transmission range (μm)	8.4
Softening Temperature (°C)	1797

**Table 2:** Comparison of sapphire hole drilling using the Bessel beam trepanning technique with previous results in the literature.

Parameters	Present Work	Reference [7]	Reference [11]
Material	Monocrystalline Sapphire (c**-**cut)	Monocrystalline Sapphire (c**-**cut)	Monocrystalline Sapphire (c**-**cut)
Beam	**Bessel**	Gaussian	Gaussian
Pulse duration	Picosecond (6 ps)	Femtosecond (300–500 fs)	Picosecond (0.8 ps)
Sample thickness	430 μm	300 μm	430 μm
Hole diameter	**≈100 μm**	≈1 mm	≈400 μm
Machining technique	Trepanning with Kapton Polyimide Tape	Bottom—Up Ablation with Spiraling	Bottom—Up Ablation with Spiraling
Tapering angle	<5°	<3°	<2°–<5°
Z-axis translation	**No**	Yes	Yes
